# Microfluidic vortex focusing for high throughput synthesis of size-tunable liposomes

**DOI:** 10.1038/s41467-022-34750-3

**Published:** 2022-11-16

**Authors:** Jung Yeon Han, Joseph N. La Fiandra, Don L. DeVoe

**Affiliations:** 1 Department of Mechanical Engineering, University of Maryland, College Park, MD USA; 2 Fischell Institute for Biomedical Devices, University of Maryland, College Park, MD USA; 3 Fischell Department of Bioengineering, University of Maryland, College Park, MD USA; 4 Present Address: Department of Bionanotechnology, Gachon University, Seongnam-si, South Korea

**Keywords:** Drug delivery, Microfluidics, Design, synthesis and processing

## Abstract

Control over vesicle size during nanoscale liposome synthesis is critical for defining the pharmaceutical properties of liposomal nanomedicines. Microfluidic technologies capable of size-tunable liposome generation have been widely explored, but scaling these microfluidic platforms for high production throughput without sacrificing size control has proven challenging. Here we describe a microfluidic-enabled process in which highly vortical flow is established around an axisymmetric stream of solvated lipids, simultaneously focusing the lipids while inducing rapid convective and diffusive mixing through application of the vortical flow field. By adjusting the individual buffer and lipid flow rates within the system, the microfluidic vortex focusing technique is capable of generating liposomes with precisely controlled size and low size variance, and may be operated up to the laminar flow limit for high throughput vesicle production. The reliable formation of liposomes as small as 27 nm and mass production rates over 20 g/h is demonstrated, offering a path toward production-scale liposome synthesis using a single continuous-flow vortex focusing device.

## Abstract (web summary)

Liposomes are widely used in pharmaceuticals yet trade-offs between uniform size and mass production, limit production and application. Here, the authors report on the design of a microfluidic vortex focusing microfluidic technique which can mass produce liposomes with controlled size and low variability.

## Introduction

The use of liposomes as nanoscale drug carriers for the controlled delivery of therapeutic agents has been widely harnessed for applications in cancer, infectious diseases, immune modulation, vaccine delivery, and beyond^1–5^. Liposomal nanoparticles support the encapsulation of the full range of hydrophilic, amphipathic, and lipophilic drug compounds within unilamellar lipid vesicles that protect the loaded drugs from degradation by the mononuclear phagocytic system (MPS) or endogenous enzymes^2,6–10^, and liposome properties may be modified during or after vesicle formation to engineer desirable biodistribution profiles via targeted cell delivery, thereby addressing issues of poor bioavailability, low plasma solubility, non-specific targeting, and high clearance rate often associated with free drug agents^1,3,10,11^. Lipidic nanomedicines have achieved wide success since the initial introduction of liposomal doxorubicin nearly 3 decades ago^12^. There are currently more than 20 liposomal nanomedicines approved for clinical use, including at least 14 for cancer treatment, and well over 100 clinical trials using lipid-based nanoparticles have been conducted in the past 5 years^13–15^.

The efficacy and toxicity of liposome-based drug delivery systems are known to be strongly influenced by liposome size. Smaller vesicles generally exhibit more uniform pharmacodynamic characteristics^4^ and offer improved bioavailability through the enhanced permeability and retention (EPR) effect^5^ which allows smaller liposomal nanoparticles to exhibit increased accumulation within tumors due to higher vascular permeability within these tissues^10,16,17^. Liposomes below approximately 100 nm can pass the blood–brain barrier^18–20^, while vesicles in the 30–40 nm range have been shown to yield significantly enhance transdermal transport^21^. Liposome size also affects blood circulation time, biodistribution, cell uptake, subcellular localization, and targeting efficiency^4,9,16,22^. Size-dependent liposomal drug toxicity has also been reported, with higher toxicity resulting from larger liposomes^9,23^ due to their increased retention in healthy tissues. Thus, tuning liposome size to the desired range while maintaining low polydispersity is essential to optimizing nanomedicine performance.

Given the importance of particle size to both the pharmacokinetics and pharmacodynamics of liposomal nanomedicines, a central challenge to the clinical translation of liposomal drugs is the need for technologies capable of generating vesicles with tight control over size and composition while also providing the throughput necessary to support all stages of the nanomedicine development and manufacturing pipeline^24–26^. Liposomes form through a self-assembly process driven by thermodynamics in combination with the free energy interactions and geometrical effects of the constituent lipid molecules^27–30^. Self-assembly can occur through the growth of small lipid fragments that transform into closed spherical vesicles as lipid solubility of the surrounding solution is reduced^28,30,31^, or by fragmenting large lipid structures that can reform into smaller unilamellar vesicles in a low solubility environment^22,30,32^. Both approaches are typically performed using batch scale process techniques such as solvent injection^33^, detergent removal^34^, membrane extrusion^22^, or high pressure extrusion^35,36^, combined with sonication^37^ or freeze/thaw cycling to reduce the final vesicle size^32,38^. While batch processing can be performed at high throughput, these methods offer limited control over vesicle size, and tend to yield liposome populations with high polydispersity. Furthermore, migrating between manufacturing scales during the different phases of nanomedicine research and production is challenging, and presents a barrier to the development process.

Continuous-flow microfluidic techniques have emerged as powerful alternatives to batch processing by improving control over the microenvironment during lipid self-assembly. In the microfluidic flow focusing method, lipids dissolved in a water-miscible polar solvent are injected into a microfluidic junction with aqueous buffer sheathing the lipid solution and hydrodynamically focusing the lipid solution into a narrow sheet^31,39–42^. Diffusive transport of solvent and water in the laminar flow environment rapidly reduce lipid solubility during focusing to promote vesicle self-assembly^43^. Due to the small lateral length scales of the focused lipid stream, which can be adjusted by changing the buffer:lipid flow rate ratio, smaller liposomes with decreased polydispersity can be achieved using this technique^29,39,41^. Another microfluidic liposome synthesis method employs rapid mixing to achieve a rapid change in solubility and small diffusive length scales through increased interfacial area in a binary fluid system^44–47^. Rapid mixing is achieved using periodic microstructures including herringbone pattern^44,48–50^, baffles^51,52^, or toroidal^25^ or twisted^26^ microfluidic channels to generate localized chaotic advection at high flow velocity, with ideal Reynolds numbers reported to be in the range of 80–100^45,47^. Although microfluidic mixers can be simpler to operate than flow focusing devices, the resulting liposome populations tend to exhibit higher size variance and a more limited size range. While the continuous-flow nature of these microfluidic techniques eliminates the need for multiple handling steps associated with batch methods, the small microchannel dimensions and laminar flow requirements constrain the throughput of the technology. Various modified flow focusing^24,53–55^ and micromixer^26^ designs have been developed to address this limitation, but with reduced size controllability and higher polydispersity observed due to the larger geometries required to support the increased buffer and lipid flow rates.

In this paper, we introduce microfluidic vortex focusing (MVF) as a technique for the production of monodisperse lipid vesicles with tunable size control while operating at high bulk flow rates to enable high throughput liposome production. The technique utilizes an axisymmetric hydrocyclone flow cell to generate a vortical flow field at Reynolds numbers approaching the laminar limit. Hydrocyclone technology, originally developed for continuous-flow particle separations, employs an inverted conical chamber with a tangential sample inlet adjacent to the cone base to generate a rotating fluid vortex that entrains particles within size-dependent streamlines, such that smaller particles are routed to an upper axial outlet at the base of the inverted cone while larger particles are routed to a lower axial outlet at the cone apex^56,57^. Miniature hydrocyclones with critical dimensions ranging from several millimeters^58–60^ to several hundred micrometers^61^ have recently been explored to reduce the achievable particle cut size, including efforts by our own group to develop small-scale hydrocyclones by additive manufacturing^62^. Here we employ a miniature cyclone with 300 µm critical dimensions to achieve nanoscale liposome synthesis by modifying the lipid sample and buffer flow paths within the device. While traditional hydrocyclones employ a single tangential inlet for the solute-laden sample stream and a pair of upper and lower outlets aligned to the conical axis, liposome synthesis is achieved by employing the tangential inlet to introduce aqueous buffer and the upper fluid port to inject solvated lipids, with the resulting liposome product collected at a single lower axial outlet. The tangential buffer inlet serves to generate a rotational flow of aqueous solution sheathing the central lipid stream, with liposome self-assembly occurring through a combination of hydrodynamic focusing and rapid mixing within the vortical flow. Vesicle formation within the cyclone chamber is a kinetic process controlled by the local solvent polarity. As lipid solubility decreases due to a combination of solvent convection, lipid advection, and solvent/lipid co-diffusion, the amphiphilic lipid molecules spontaneously form planar disc-like micelles. These intermediate structures grow in a reaction rate limited process until the line energy associated with the exposed hydrophobic lipid tails overcomes the elastic energy required to form spherical vesicles, at which point membrane closure becomes energetically favorable^63,64^. Because the micelle growth rate and the elastic energy at closure are both dependent on lipid solubility, a sharp temporal solubility gradient can enable the formation of smaller liposomes by limiting the intermediate lipid structure growth time. In the cyclonic flow cell, stretching and folding of the fluid interface under the influence of the vortical flow field contributes to rapid mixing of the miscible aqueous and lipid streams^65^. This aspect of the mixing process is similar to other vortex mixers explored for liposome synthesis^66–69^, in which both lipid and buffer are injected tangentially into a mixing chamber to generate chaotic advection patterns in a manner similar to herringbone or toroidal micromixers. Compared with mixing by conventional ethanol injection^70^, vortex mixing can yield enhanced mixing rates and improved control over liposome size. However, as with microfluidic chaotic advection mixers^44–47^, liposome polydispersity tends to be high due to spatial variations across the mixing zone, with reported polydispersity index (PDI) values typically above 0.2 regardless of flow rate ratio^67,69^.

In this work, microfluidic vortex focusing is shown to overcome the limitations of standard vortex mixers by sheathing the lipid stream with an outer flow of aqueous buffer in a manner similar to microfluidic flow focusing, spatially constraining the mixing zone and significantly reducing the diffusive length scale during vortex mixing. To achieve the complex 3D geometry required to generate the required focusing zone and vortical flow field, devices are fabricated via high resolution additive manufacturing by stereolithography (SLA) utilizing a digital light processor (DLP) for layer-by-layer pattern generation. Vortex formation and solvent transport within the system are investigated through numerical simulations, and fabricated devices are used to experimentally evaluate liposome formation under different operating conditions and lipid compositions. The resulting devices achieve reliable liposome synthesis and narrow size distributions, with vesicle diameters ranging from 61 nm to 127 nm for a lipid composition without polyethylene glycol (PEG), and as small as 27 nm when introducing a PEG-conjugated lipid in the mixture. The devices support efficient and repeatable operation at mass production rates that can exceed 20 g/h without sacrificing size uniformity.

## Results and discussion

### Device design and modeling

**Fig. 1:**
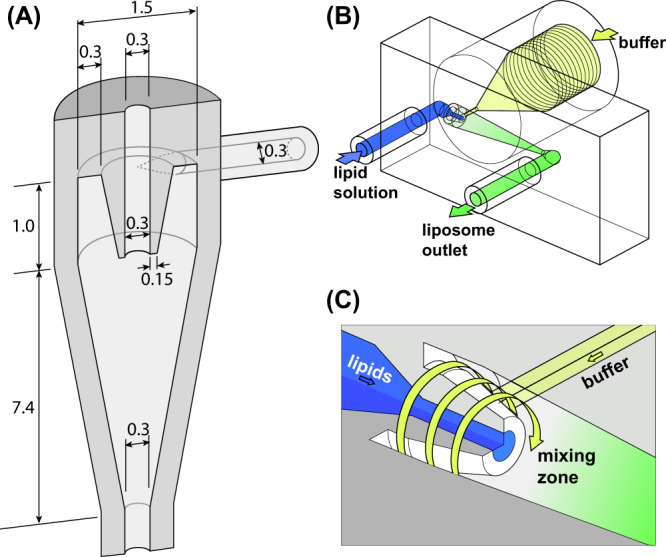
Microfluidic vortex focusing device design and operation. **A** Schematic of the fluidic structure for vortex generation and flow focusing (all dimensions in mm). **B** The MVF device design consists of two inlets conjoining at the annular junction, a conical mixing region, and an outlet. **C** Magnified view on the annular junction.

In the established hydrodynamic focusing method for liposome formation, a sharp solubility gradient is generated by narrowing the lipid stream to reduce the length scale for lateral diffusion of solvent, lipid, and aqueous buffer within the system. Similarly, chaotic advection micromixers leverage rapid laminar mixing to reduce the effective diffusion length scales for control over vesicle size. In the present work we explore a platform that combines hydrodynamic focusing and vortex-enhanced mixing in a single process. An overview of the device design is depicted in Fig. 1A. To generate the vortical flow field during focusing, aqueous buffer is injected tangentially into the upper annular region of an axisymmetric chamber, resulting in a spiral flow path that continues into the lower conical mixing chamber. Lipid solution is injected through an axial inlet within the annular vortex generation zone and emerges into the center of the mixing chamber sheathed by the rotating buffer flow, which transfers rotational momentum to the lipid solution. To promote efficient focusing, the inner surface of the annular structure is tapered to minimize geometric discontinuity at the fluid junction. After mixing within the conical chamber, the combined flow exits through a lower fluid port for collection. A schematic of the full device design including inlet and outlet ports is presented in Fig. 1B, with a detailed view of the vortex generation zone and focusing junction shown in Fig. 1C.

Fluid mixing occurs when multiple fluid elements are brought into contact, allowing scalar concentrations within each volume to diffuse across the interface^71^. In the case of a 2D steady vortex flow, local shear results in fluid elements located on different streamlines to separate, thereby stretching the fluid interface and folding adjacent elements around one another^65^. For the 3D vortex focusing system, hydrodynamic focusing of the lipids during injection into the vortex flow serves to shrink the initial axial cross section of the lipid stream, thus minimizing the radial length scale of the stretched fluid volumes. Taken together, this process serves to reduce the diffusion length scale and mixing times for the polar and aqueous solvents as well as the lipid solutes themselves.

**Fig. 2:**
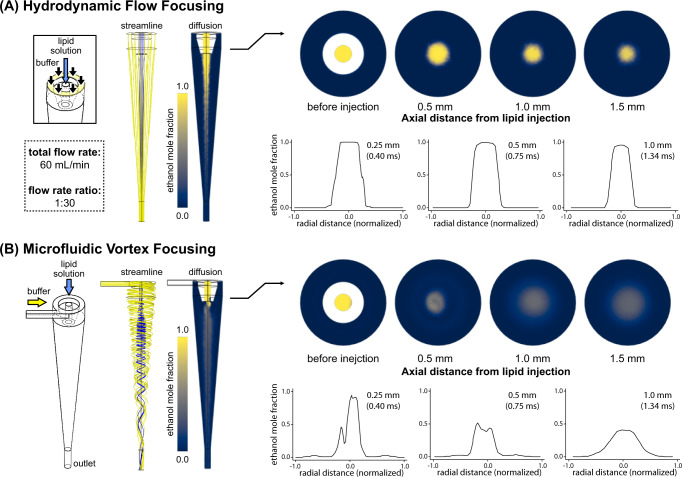
Numerical simulations of water/ethanol co-flows. Simulated flow streamlines and ethanol concentration profiles are presented for numerical simulations of **A** hydrodynamic focusing and **B** microfluidic vortex focusing. For each case, details of the radial solvent profiles are shown at increasing distance from the lipid injection point. A total flow rate of 60 mL/min and flow rate ratio of 1:30 (ethanol:water) was employed in both simulations.

To study the combined mixing and focusing process, a numerical model was developed to reveal the distribution of solvent during both vortex focusing and hydrodynamic flow focusing, with results summarized in Fig. 2. In this numerical study, hydrodynamic focusing was performed using the same device geometry employed for vortex focusing, but with the aqueous buffer injected axially instead of tangentially into the annular region to prevent vortex formation. For both device configurations, the evolution of the solvent concentration profile is a critical factor in predicting the final liposome size. Vesicle formation begins when reduced lipid solubility leads to the formation of small bilayer fragments that continue to grow in a rate-limited process until vesicle enclosure becomes energetically favorable. Here we infer an approximate threshold solvent concentration at which vesicle formation begins from a prior study of DMPC aggregation as a function of ethanol concentration^72^. In this work, which employed light scattering to evaluate particle anisotropy as the solvent concentration was reduced, a sharp decrease in dissymmetry and increase in depolarization was observed at a solvent mole fraction of 0.5, consistent with the formation of disk-like structures around this value of ethanol concentration. Based on this observation, we assume a mole fraction of 0.5 as an appropriate threshold below which vesicle formation begins to occur in our experiments. As revealed in Fig. 2A for the case of hydrodynamic flow focusing, the peak ethanol concentration remains at a value near unity at a point 1.5 mm downstream of the fluid junction, and only reaches the selected threshold value of 0.5 after travelling an axial distance of 3.1 mm. In contrast, during MVF the peak ethanol mole fraction drops to approximately 0.5 within 500 µm of the focusing junction (Fig. 2B). Additional details of the simulated mixing length scales during microfluidic vortex focusing and hydrodynamic focusing are presented in Supplementary Fig. 2. For the bulk buffer and lipid flow rates used in this study, the threshold concentration during vortex focusing is reached within 0.75 ms after entering the mixing zone, compared with 2.50 ms for the case of hydrodynamic flow focusing. The significantly faster mixing associated with the vortex focusing process limits the time available for lipid fragment growth, and thus is expected to enable the synthesis of smaller vesicles.

### Device fabrication

**Fig. 3:**
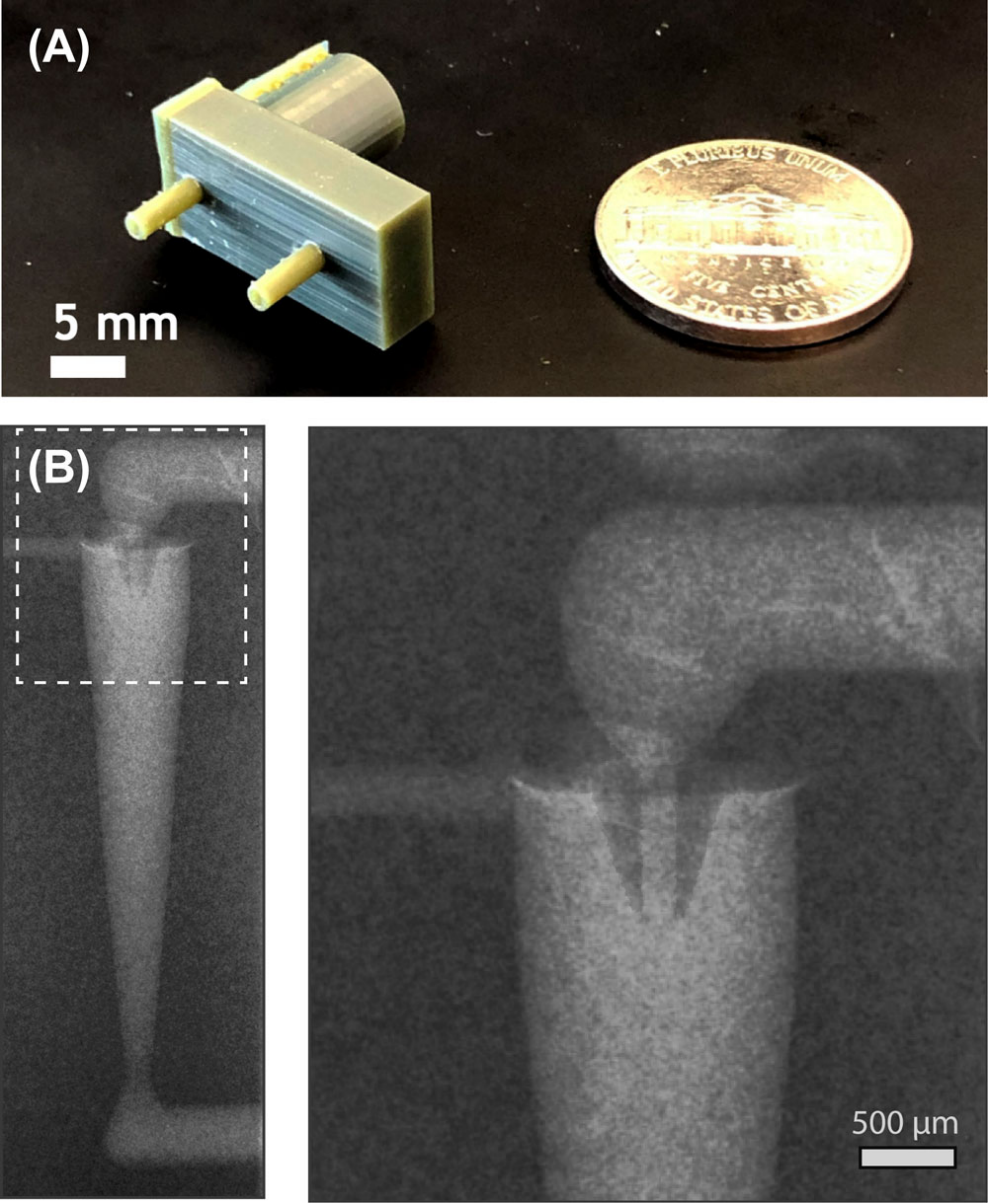
Fabricated vortex focusing device produced by SLA-DLP. **A** Image of a printed device showing inlet and outlet ports. **B** Contrast-enhanced micro-CT image revealing the internal structure of the fluidic junction where solvated lipids are injected into a rotational flow of aqueous buffer.

A fabricated MVF device together with details of the internal structure imaged by microtomography are presented in Fig. 3. Print orientation was found to be play an important role in realizing the tapered annular structure that serves as a nozzle for the lipid inflow. The axial direction of the MVF design was aligned perpendicular to the print stage, with the upper ceiling of the MVF design facing the print stage, allowing the thicker base of the tapered annular structure to be formed before patterning the tapering geometry. Another important factor is the symmetry of the annular nozzle. Since mixing occurs at the junction of the annular vortex generation zone and the annular lipid inlet, nozzle asymmetry may dramatically affect the flow and mixing profiles during focusing. While orienting the devices perpendicular to the stage during printing helped to reduce asymmetry, nozzle deformation was found to be further minimized by defining a fixed thickness for the nozzle tip, rather than allowing the geometry to taper to a point. A tip thickness of 150 μm was found to be the minimum dimension that could be reliably formed for the particular printing tool and resin used in this work. During process optimization, we sought to minimize the lipid injection channel diameter and wall thickness with the goal of reducing the radial mixing length scale during liposome formation. While channel dimensions as small as 150 µm were investigated, a diameter of 300 µm was selected for the final devices since smaller ports were routinely found to be closed prior to the final development step. Maintaining a maximum nozzle length of 1 mm was also found to be critical, with longer nozzles frequently resulting in warping or clogging (Supplementary Fig. 3). Finally, the light intensity during stereolithography was carefully optimized to improve device geometry and performance. Increased light intensity results in greater photopolymerization and improved mechanical stability of the resulting prints, but degrades the effective voxel resolution. Conversely, decreased light intensity can enable smaller features at the risk of yielding mechanically unstable prints due to insufficient crosslinking. For the device geometry explored here, exposure intensity was limited to 97% of the nominal instrument level. Under the optimized processing conditions, device yield was approximately 50%, with full or partial clogging of the lipid channel being the primary failure mode. Surface roughness within the vortex focusing chamber was measured by optical profilometry after cutting open the chamber using a low speed saw, with average roughness values of R_a_ = 1.32 µm and R_a_ = 0.45 µm observed in the axial and radial directions, respectively.

Characteristics of the resin used for device fabrication can also impact device performance. To support liposome synthesis, the material must be compatible with the solvent used as a lipid carrier, and must offer sufficient rigidity and mechanical strength to avoid deforming or breaking during high pressure operation. To ensure compatibility with ethanol as the lipid solvent, an acrylic-based photosensitive resin (HTM140) was selected. This resin demonstrated excellent chemical stability in our tests, with no dissolution, deformation, or crack development following 7 days of immersion in ethanol. Secondary UV exposure to fully polymerize the resin after development is essential to prevent solvent-induced mechanical failure such as cracking due to the presence of unreacted monomer, oligomers, or low molecular weight polymers within the solidified resin. The selected resin is also a relatively rigid and strong material with the tensile strength of 56 MPa according to the manufacturer, and has been previously demonstrated for high-pressure applications including gas chromatography columns^73^.

### Liposome synthesis

Particle diameter and size distribution are important parameters for controlling therapeutic effect and safety for all nanocarrier systems^74^. To emphasize the importance of controlling vesicle size and polydispersity for liposomal nanomedicines, consider a liposome population with known mean diameter and polydispersity index (PDI), defined as the particle size variance normalized by the square of the mean diameter^75^. The volume of hydrophilic drug encapsulated within the liposome core for a given vesicle size range can be determined by integrating the product of the distribution probability density function and size-dependent particle volume. Given a log-normal particle size distribution^76,77^ with location and scale parameters derived from mean diameter and PDI values^78^, a population with a mean diameter of 100 nm and PDI of 0.2 is found to have approximately 80% of the total drug encapsulated by the liposomes retained within particles larger than 100 nm, i.e., within a size range where delivery to the targeted tissues may not be optimal and accumulation in healthy organs can occur. In contrast, reducing the size to 80 nm and PDI to 0.05 significantly reduces the drug associated with these larger particles to below 28%. A similar analysis applies for the case of hydrophobic drug intercalated within the liposome membrane, where drug amount scales approximately with membrane area. In this case, 67% of the drug is found to be retained in vesicles above 100 nm for the larger and more polydisperse vesicle population, compared with only 21% for the smaller and more uniform particles.

**Fig. 4:**
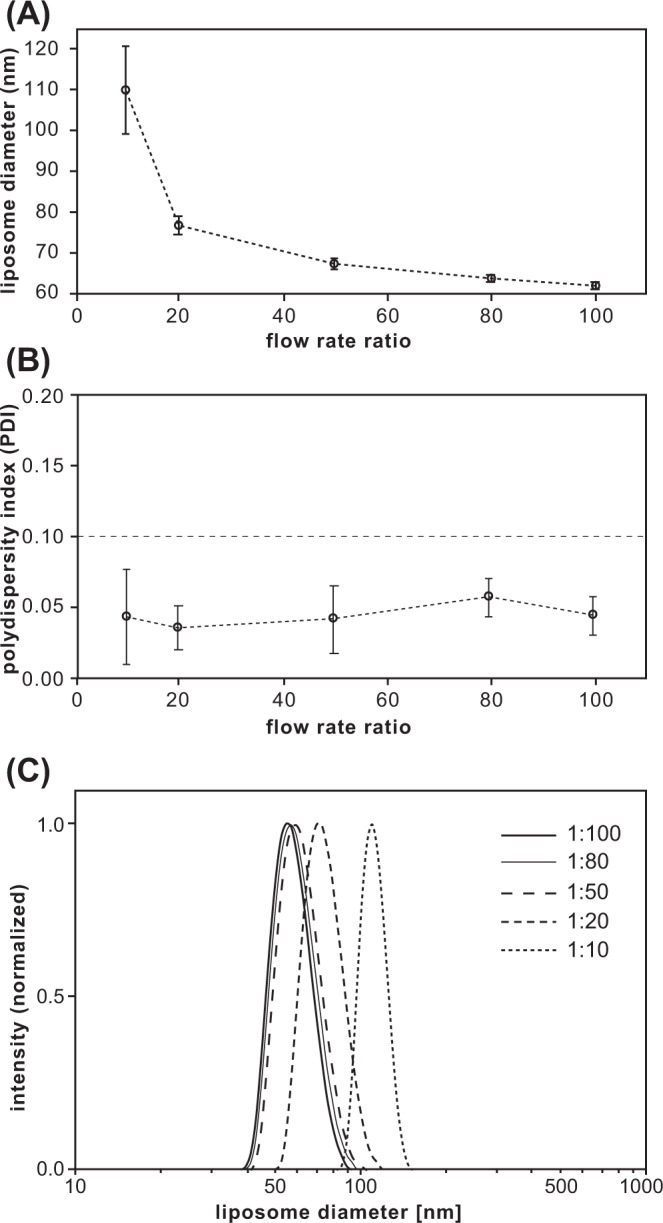
Impact of buffer:lipid flow rate ratio (FRR) on liposome size and polydispersity for a lipid mixture of DMPC:cholesterol:DCP (5:4:1 molar ratio). **A** Average liposome diameter, **B** polydispersity index, and **C** normalized size distribution plots. Total flow rate (TFR) and initial lipid concentration were fixed at 60 mL/min and 10 mM, respectively. *N* ≥ 6, error bars ± 1 SD.

As with other microfluidic liposome synthesis techniques, the size of liposomes generated by vortex focusing can be directly controlled by adjusting the relative flow rates of solvated lipid and aqueous buffer injected into the system. The impact of the buffer:lipid flow rate ratio (FRR) on liposome size and size distribution is presented in Fig. 4. Using a constant total flow rate of 60 mL/min and 10 mM lipid concentration in the injected ethanol stream, increasing the flow rate ratio over a full log from 10 to 100 led to a significant decrease in liposome size, with a minimum diameter of 61 nm at FRR = 100 (Fig. 4A). The process was highly repeatable, with minimal variation in mean diameter observed over 6 replicates for each liposome synthesis condition. The inverse relationship between FRR and vesicle size is consistent with liposome formation using both hydrodynamic flow focusing^29,39,41^ and rapid micromixing^44–47,67^. However, the vortex focusing process was found to yield low polydispersity, with an average PDI value of 0.04 and nearly constant size variance over the full range of flow rate ratios (Fig. 4B). This behavior differs from microfluidic chaotic advection mixers where PDI increases with FRR^48^. In contrast, the widely-used ethanol injection method tends to yield lower size variance at higher buffer:ethanol flow ratios^70,79^, suggesting an alternate path to controlling liposome size without requiring the use of microfluidics. However, ethanol injection systems typically require lower lipid concentrations to reduce vesicle size, which has been shown to yield a concomitant increase in size variance^80^. Overall, the simultaneous reduction of liposome size and polydispersity remains challenging for established liposome production techniques, particularly at higher system throughput. Defining a production figure of merit (Q) as the inverse of the product of liposome diameter(d) and polydispersity, i.e., Q = d^−1^PDI^−1^, we find that Q values for the MVF platform are generally in the range of 0.1–0.4, whereas reported data for both ethanol injection^81–87^ and chaotic advection micromixing^25,48,50,88–91^ yield Q values below 0.15 (Supplementary Fig. 4). Note that because unsaturated lipids increase bilayer packing defects that can significantly lower the membrane bending energy and impact the liposome formation process, the data presented in Supplementary Fig. 4 is limited to empty liposomes formed using saturated lipids such as DMPC as their primary component.

As with other vesicle formation techniques employing flow focusing, a potential limitation of the MVF process is the need for high FRR values to reduce vesicle size. Increasing the flow rate ratio introduces the need to concentrate the resulting liposome solutions for clinical use, and may also impact drug encapsulation efficiency. In practice, all liposome synthesis techniques including other microfluidic methods require downstream processing for buffer exchange, filtration, and concentration adjustment^92^. To concentrate dilute suspensions of larger liposomes, conventional ultracentrifugation is commonly employed^33^, while smaller nanoliposomes may be efficiently concentrated by centrifugal filtration^93–96^ using a filter element with an appropriate cutoff size that can also serve to remove solvent and free drug from the final liposome suspension. Evaluating the impact of high FRR operation on post-processing requirements will be needed to advance the MVF technology for liposomal drug production. Similarly, achievable drug encapsulation efficiency is an additional consideration requiring further study for the MVF platform. As with other flow-focusing methods, operation at higher FRR values is expected to result in lower encapsulation efficiencies for hydrophilic agents introduced with the aqueous sheath flow, and the impact of the coupled vortex mixing and focusing process on the encapsulation of both hydrophilic drugs in the aqueous phase and hydrophobic drugs added to the lipid stream is not presently known.

**Fig. 5:**
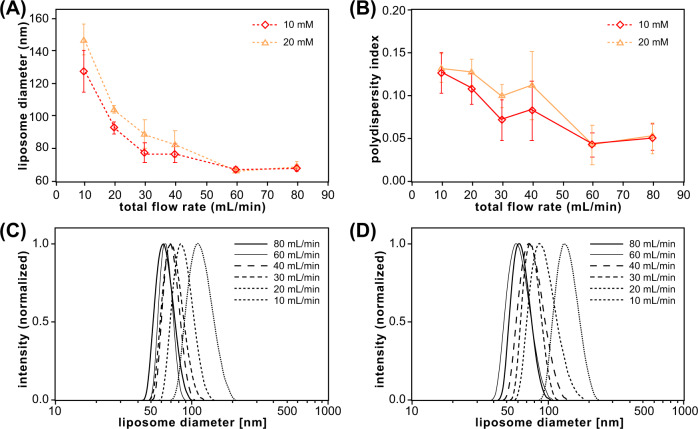
Liposomes synthesized using a MVF device over a range of TFR values at two different total lipid concentration (TLC) values and a fixed FRR of 50 for a lipid mixture of DMPC:cholesterol:DCP (5:4:1 molar ratio). **A** Average diameter and **B** polydispersity index of produced liposomes. *N* ≥ 9, error bars ±1 SD. **C** Normalized size distribution plots of liposomes synthesized at TLC values of 10 mM (left) and 20 mM (right).

When operating the vortex focusing device at constant FRR, the overall liposome production throughput may be enhanced by increasing the total flow rate (TFR) of the combined lipid and buffer flows through the system. The impact of TFR on liposome size distribution is presented in Fig. 5. With the flow ratio held at a constant FRR value of 50, a strong inverse relationship between total flow rate and both liposome size and PDI was observed (Fig. 5A, B). This differs from the reported behavior of other microfluidic techniques including both hydrodynamic focusing^24,97^ and chaotic advection mixing^25,48,98,99^ where vesicle size and polydispersity are generally insensitive to TFR. This difference may be explained by the influence of vortical flow on mass transport within the MVF platform. At higher aqueous buffer flow rates, the rotational flow velocity surrounding the injected lipid solution increases, thereby enhancing mixing during the focusing process (Supplementary Fig. 5). The same behavior is not observed in either flow focusing or chaotic advection since the mixing streamlines in these techniques are invariant with total flow rate.

**Fig. 6:**
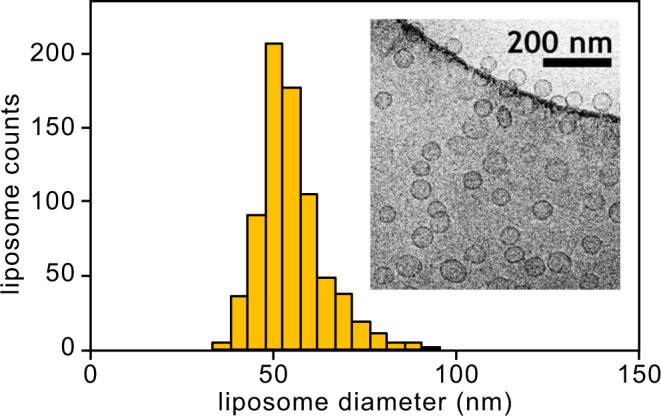
Histogram of liposome diameters measured by cryo-TEM imaging. Liposomes were synthesized at a TFR of 80 mL/min, FRR of 50, and TLC of 10 mM using a lipid mixture of DMPC:cholesterol:DCP (5:4:1 molar ratio).

The impact of lipid concentration on liposome formation is also presented in Fig. 5. A small increase in both mean diameter and PDI was observed when raising the concentration of lipids in the feed stream from 10 mM to 20 mM. Measured size distributions for the particles generated under both lipid concentrations are provided in Fig. 5C. A representative size distribution generated by direct characterization of a liposome sample imaged by Cryo-TEM is shown in Fig. 6 for comparison with the DLS data.

Long term storage of liposomes is an important consideration for nanomedicine applications. Structural instability driven by thermodynamic perturbations can lead to degradation and structural reorganization of the vesicles. To evaluate the colloidal stability of vesicles generated by vortex focusing, 3 selected liposome populations formed under different total flow rates and flow rate ratios were stored at 4 °C for 99 days, with size distributons measured before and after storage. During this time period no detectable change in mean particle size or size variance was observed for any of the samples (Supplementary Fig. 6).

**Fig. 7:**
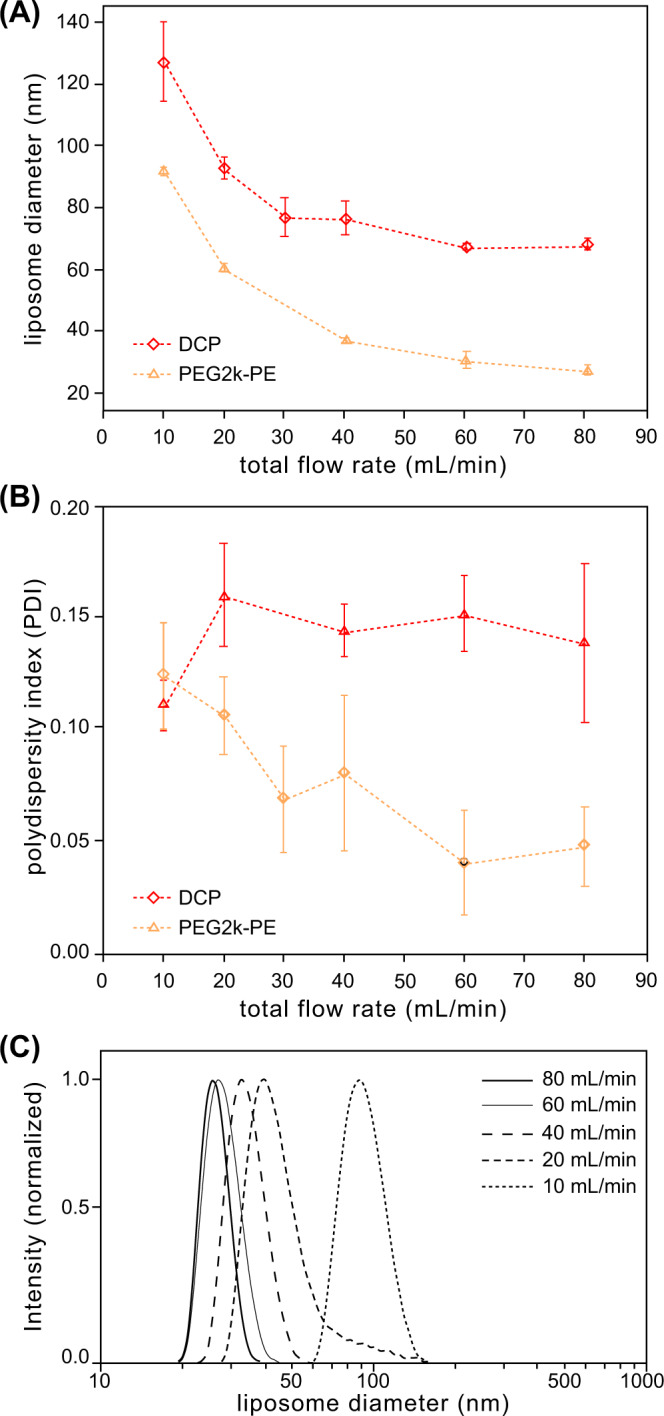
Impact of PEG-lipids on vesicle size distributions. Liposomes were prepared with either DMPC:cholesterol:DCP or DMPC:cholesterol:PEG2k-PE at a 5:4:1 molar ratio. **A** Mean diameter and **B** polydispersity index of both liposome populations at different total flow rates. **C** Normalized size distribution plots of the PEGylated liposomes. For all cases FRR = 50 and total lipid concentration = 10 mM. *N* ≥ 6, error bars ± 1 SD.

Liposome surface modifications can also impact pharmacokinetic properties. In particular, the attachment of PEG to the outer liposome surface allows the nanoparticles to avoid recognition by the MPS^8^, enabling longer blood circulation times, improved bioavailability, and higher levels of accumulation in tumor tissues^9,30^. Because the presence of large PEG molecules imposes steric effects during liposome formation, it is desirable to understand the relationship between PEG content and vesicle size for this important class of nanocarrier. The inclusion of PEG-conjugated lipids during nanoparticle formation is known to stabilize the resulting particles. While an inverse relationship between particle size and both PEG concentration and PEG chain length has been reported for the case of solid lipid nanoparticle nucleation^100^, no significant change in liposome size was observed when adding increasing concentrations of PEG-lipids during liposome synthesis by hydrodynamic flow focusing^101^. To investigate this issue for the vortex focusing process, a high concentration (10 mol%) of PE conjugated to PEG-2000 was introduced to the lipid feed solution before operating the vortex focusing device at FRR = 50 and 10 mM lipid concentration while varying the total flow rate. The resulting measurements of vesicle size are presented in Fig. 7A, with corresponding polydispersity shown in Fig. 7B. The presence of PEGylated lipids during vesicle formation was found to result in a significant reduction in vesicle size at all flow rates, with a maximum decrease of nearly 60% at a total flow rate of 80 mL/min, leading to a minimum mean vesicle diameter of 27 nm. However, polydispersity for the PEG-lipid mixture remained nearly invariant with flow rate, with an average PDI of 0.14 over the tested range. Because the final liposome diameter depends on the time scale over which lipid bilayer fragments are allowed to grow before the surrounding medium reaches a polarity limit at which vesicle formation becomes energetically favorable^41^, the smaller PEG-lipid vesicles may reflect slower growth kinetics of the intermediate bilayer structures due to lower diffusivity of the PEG-lipid conjugates.

### High-throughput operation

**Fig. 8:**
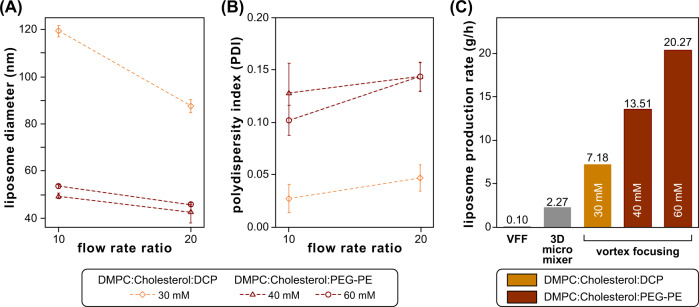
High production rate synthesis of liposomes by microfluidic vortex focusing. **A** Liposome characteristics for DMPC:cholesterol:DCP at 30 mM total lipid content, and DMPC:cholesterol:PEG2k-PE at 40 mM and 60 mM (error bars ±1 SD). **B** Comparison of the resulting mass production rates with other high throughput microfluidic liposome synthesis technologies, namely vertical flow focusing (VFF)^24^ and 3D micromixing^26^. FRR was maintained at 10 for the MVF experiments. All lipid mixtures employed a molar ratio of 5:4:1 molar ratio.

A central advantage of the vortex focusing technology is the ability to generate size-controlled liposomes by taking advantage of simultaneous flow focusing and vortical mixing, while operating at bulk flow rates that can be significantly higher than conventional hydrodynamic flow focusing or chaotic advection micromixer platforms. Maximum flow rates are dictated by the need for laminar flow conditions within the flow cell to maintain efficient focusing. Taking the main chamber radius as the critical dimension for the system, the resulting Reynolds number is found to approach the laminar limit when operating at a total flow rate of 60 mL/min, and begins to enter the transitional regime at the highest tested flow rate of 80 mL/min. Thus, higher flow rates cannot be employed to improve throughput without inducing turbulent flow, destabilization of the focusing zone and leading to higher polydispersity and an overall reduction in size control. However, increasing the lipid feed concentration represents an alternate path to higher mass production rates independent of lipid and buffer flow conditions. To explore this option, liposomes were synthesized for each lipid mixture with concentrations approaching the lipid solubility limit while operating at the maximum flow rate of 80 mL/min. For the DCP-based lipid mixture, solubility in dehydrated ethanol was maintained for lipid concentrations up to 30 mM, while the PEGylated lipid mixture remained soluble up to 60 mM. As presented in Fig. 8, mass production rates as high as 7.2 g/h were achieved for the DCP-based liposomes, while the higher solubility limit of the PEGylated lipid mixture enabled a maximum rate over 20 g/h. This throughput is more than 50 times higher than previously demonstrated for high aspect ratio hydrodynamic focusing^24,97^, and nearly an order of magnitude higher than emerging chaotic advection mixing platforms developed for high throughput operation^26^. For all tested flow rates, the full incorporation of lipids into the desired bilayer vesicles was inferred from the lack of peaks associated with smaller micelles or larger lipid aggregates in the resulting light scattering data.

In conclusion, the microfluidic vortex focusing technique combines the advantages of hydrodynamic flow focusing and chaotic advection mixing to enable size-tunable liposome generation while operating at high levels of lipid flux. Significantly, liposome production rates demonstrated in this work are within the range of pilot and manufacturing scale liposomal drug production lines, typically on the order of 1–60 g/h^92,102,103^. Like other microfluidic liposome synthesis technologies, vortex focusing is a continuous-flow process that may be operated with minimal labor and infrastucture, while avoiding sequential batch processing steps associated with conventional methods. However, unlike established microfluidic techniques, vortex focusing provides greater control over both the mean size and size variance for the resulting vesicles, particularly while operating at higher lipid mass transport rates. Compared with prior our work on both low-throughput^31,40,41,43,104^ and high-throughput^24,97^ hydrodynamic flow focusing devices, liposome synthesis by microfluidic vortex focusing was also found to yield substantially higher levels of repeatability, with negligible variation in mean size and PDI observed when operating devices under identical conditions over multiple days. Because the vortex focusing devices are manufactured using a proven SLA-DLP 3D printing process, their fabrication is cost-effective and reliable, and can be readily implemented without specialized microfabrication equipment or training, making the technology available to a wider range of potential users. Finally, we note that while the technique is applied to liposome production in this work, vortex focusing may also provide advantages for other high-throughput nanoparticle synthesis applications such as solid lipid nanoparticles or inorganic nanoparticles where particle formation can be controlled by taking advantage of rapid diffusive and convective transport within the continuous-flow system.

## Methods

### Device fabrication

Devices were fabricated using a high resolution SLA-DLP process leveraging our work on 3D printed microscale hydrocyclones for particle separation and concentration^62^. Overall device dimensions were identical to this prior study, including total chamber diameter and length of 1.5 mm and 8.4 mm, respectively, vortex formation gap of 300 µm, and inlet/outlet channel diameters of 300 µm. To minimize the fluid dead volume during lipid focusing, the lipid injection channel was tapered to a final tip thickness of 150 µm. A threaded port design was employed for the buffer inlet to support the use of an Upchurch F-120 fitting (IDEX Health & Science, Oak Harbor, WA) for high-pressure fluidic connection. The resulting stereolithography (STL) file was converted into a mask layer stack for SLA-DLP printing with 25 µm z-step on a Perfactory 4 DLP-SLA instrument (EnvisionTEC Inc., Dearborn, MI) using the EnvisionTEC Magics software. The stereolithography tool was equipped with a 75 mm objective lens for high-resolution printing, corresponding to a 74 mm × 46 mm printable area at 1920 × 1200 pixel resolution. The STL file was oriented to align the axial center of the MVF design perpendicular to the print stage. To optimize feature resolution, the light intensity of the tool was adjusting using a 3-stage 48-point calibration process for each run. After printing, developing was performed in a light-protected environment. The device was first rinsed with IPA using a spray bottle, then connected to a 20 mL syringe through tygon tubing equipped with a blunt needle segment inserted into an F-120 fitting. The device was immersed in an isopropanol (IPA) bath, and the syringe plunger was manually withdrawn to displace residual resin within the device with IPA. The IPA flush was continued until the withdrawn flow changed from dark green (resin color) to colorless. The device was then air-flushed to remove IPA and the fabrication process was completed by curing the printed devices using an Otoflash UV curing unit (EnvisionTEC Inc., Dearborn, MI) for 500 flashes. To determine the print quality, the internal structure of selected devices was examined by x-ray microtomography using a SkyScan 1276 X system (Bruker Scientific Instruments, Billerica, MA).

### Numerical modeling

Co-flow of water and ethanol within the MVF device was simulated numerically to evaluate the flow characteristics and identify appropriate operational conditions using COMSOL Multiphysics software (COMSOL Inc., Burlington, MA). The simulation model was designed with identical geometry as the MVF device, with the exception of the buffer inlet which was modified to have a square cross section instead of circular for efficient meshing. The binary phase of the miscible fluids was simulated using a stationary solver with second order discretization for both pressure and velocity fields at the interface of fluidic elements to account for nonlinear characteristics of the vortical two-phase flow. Both fluids were considered incompressible with a no-slip wall boundary condition applied. To simulate vortex formation, focusing, and mixing, ethanol was introduced from the lipid inlet located at the upper end of axial center while water flow entered from the inlet tangentially attached to the upper body of the MVF device. Flow characteristics were studied for varying total flow rates from 10 mL/min to 80 mL/min, and for varying water:ethanol flow rate ratios from 10 to 50. To compare MVF with the conventional hydrodynamic focusing, the similar MVF model without a tangential inlet was studied by setting the inlet for water feed to the annular ceiling of the MVF design.

### Liposome synthesis

Ethanol (200 proof) was further dehydrated by adding molecular sieve at approximately 20 wt% of ethanol and resting for at least 24 h. Lipid stock solution was prepared by adding 1,2-dimyristoyl-sn-glycero-3-phosphocholine (DMPC), cholesterol, and dicetyl phosphate (DCP) at a molar ratio of 5:4:1 in chloroform. A 1 mL aliquot of the stock solution was transferred to a glass scintillation vial and subjected to gentle nitrogen flow to evaporate chloroform and leave a thin lipid film, and the solvent was further removed from the lipid film in vacuo at room temperature for 4 h. Upon completion of solvent removal, each vial was gently flushed with nitrogen and sealed to minimize oxidation. For PEGylated liposomes, the lipid stock solution was prepared using DMPC, cholesterol, and 1,2-distearoyl-sn-glycero-3-phosphoethanolamine-N-[methoxy(polyethylene glycol)−2000] (PEG2k-PE) at the same molar ratio and following the same protocol. All lipids were purchased from Avanti Polar Lipids (Birmingham, AL), and other reagents were purchased from Thermo Fisher Scientific (Waltham, MA).

For liposome synthesis, the prepared dried lipid film was first solubilized with 1 mL of dry ethanol to yield a 20 mM lipid solution. The solution was loaded into a 1 mL syringe and connected to the lipid inlet of the MVF device, and a 60 mL syringe with 1× PBS solution was connected to the buffer inlet. Each solution was filtered thru a 0.22 μm syringe filter prior to use. While smaller liposomes were realized when using HEPES or Tris as alternate buffer solutions (Supplementary Fig. 1), PBS was employed for all experiments due to its close match to physiologic osmolarity and ionic strength as well as lack of toxicity. The MVF device was submerged into a water bath at 40 °C, then perfused with 1×PBS to remove air from the flow path. Liposomes were then synthesized by operating both syringes at the desired flow rate. To evaluate long-term liposome stability, solutions were stored in a sterilized container at 4 °C. All buffers were autoclaved prior to use, and the resulting samples were processed through 0.22 µm syringe filters.

### Liposome characterization

Size distributions for each liposome population were determined using a dynamic light scattering (DLS) instrument (Zetasizer Nano ZS, Malvern Panalytical Inc., Westborough, MA). All samples were filtered with a 0.22 μm syringe filter before being loaded into a cuvette. The chamber temperature was set to 25 °C throughout the DLS measurement. As-prepared liposomes were measured directly, and liposome samples stored at 4^o^ C for stability testing were left at the room temperature for 1 h prior to measurement. For electron microscopy, cryo-TEM samples were prepared using a Cryoplunge3 (Gatan Ametek, Pleasanton, CA) and imaged with a JEM 2100 LaB6 (JEOL USA Inc., Peabody, MA).

## Supplementary information

Supplementary Information

## Data Availability

All quantitative data generated in this study have been deposited in the Open Science Framework platform under https://osf.io/7usdc.
