# Supplementary material for: Microfluidic vortex focusing for high throughput synthesis of size-tunable liposomes
Source: Nat Commun. 2022 Nov 16;13:6997. doi: 10.1038/s41467-022-34750-3 (PMC9668976; doi:10.1038/s41467-022-34750-3)
Supplement: Supplementary file 1 — Supplementary Information [file 41467_2022_34750_MOESM1_ESM.pdf]

Supplementary Information

Microfluidic vortex focusing for high throughput synthesis of size-tunable liposomes

Jung Yeon Han[1,2,§], Joseph N. La Fiandra[3], and Don L. DeVoe[1,2,3]*

[1]Department of Mechanical Engineering, University of Maryland, College Park, MD, USA

[2]Fischell Institute for Biomedical Devices, University of Maryland, College Park, MD, USA

[3]Fischell Department of Bioengineering, University of Maryland, College Park, MD, USA

[§]Current affiliation: Department of Bionanotechnology, Gachon University, South Korea

[*]Corresponding author: ddev@umd.edu

Contents:

Supplementary Figure 1: Effect of buffer selection on liposome synthesis.

Supplementary Figure 2: Numerical simulations comparing mixing length scales.

Supplementary Figure 3: Variable SLA-DLP print quality.

Supplementary Figure 4: Comparison of MVF device performance.

Supplementary Figure 5: Numerical analysis revealing the impact of total flow rate on mixing dynamics.

Supplementary Figure 6: Evaluation of long-term colloidal stability of liposomes generated by MVF.

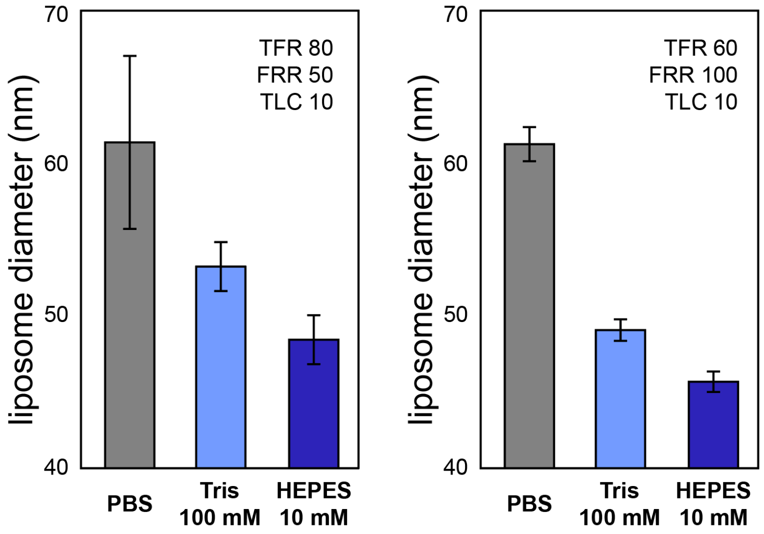

Supplementary Figure 1: Effect of buffer selection on liposome synthesis. (left) Liposomes produced using DMPC:cholesterol:DCP (5:4:1 molar ratio) at TFR 80 mL/min, FRR 1:50, TLC 10 mM, and (right) produced at TFR 60 mL/min, FRR 1:100, TLC 10 mM. Three types of buffer include 1× PBS at pH 7.5, Tris buffer at 100 mM concentration and pH 8.1, and HEPES buffer at 10 mM concentration and pH 7.5. Error bars ±1 SD.

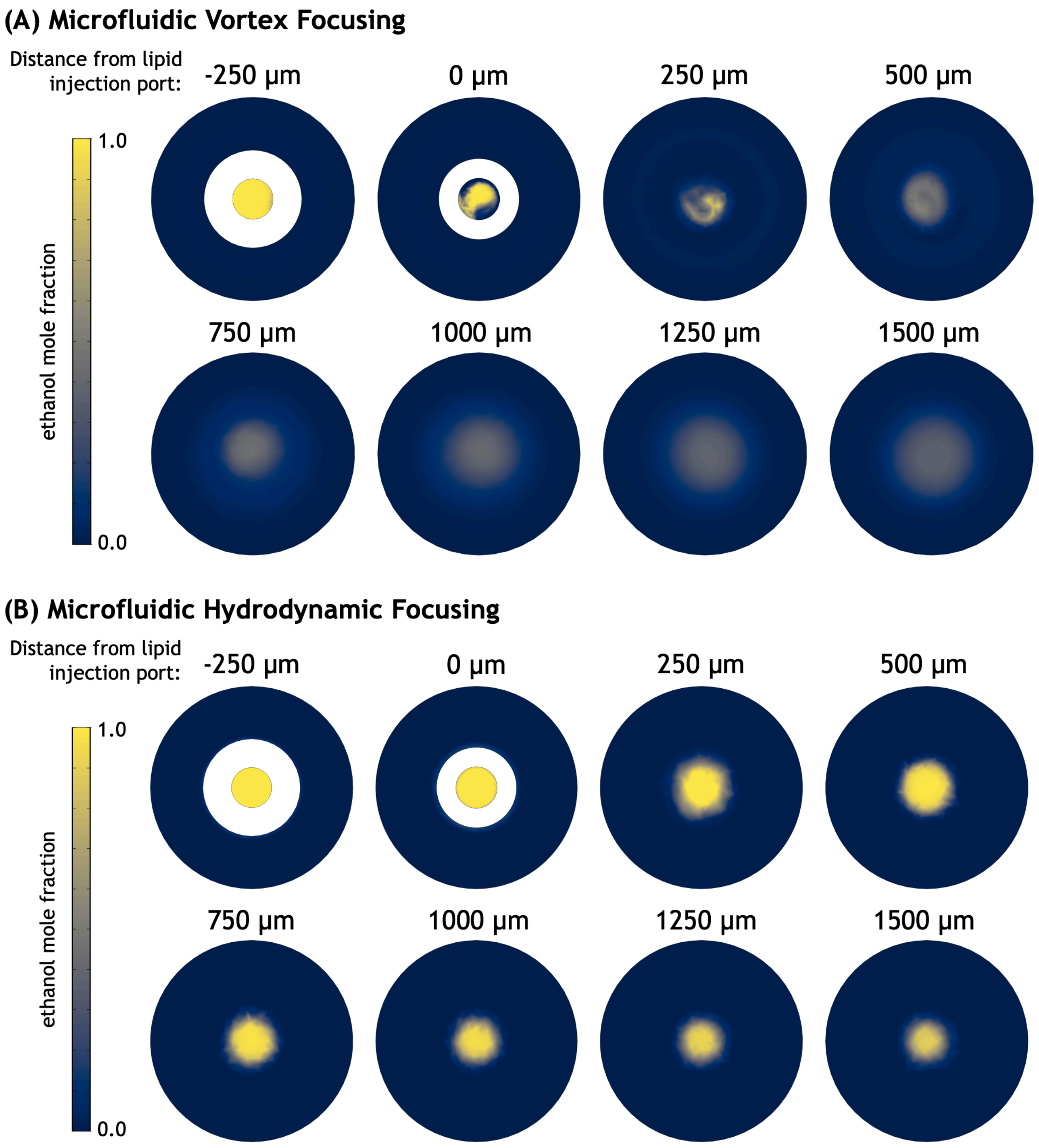

Supplementary Figure 2: Numerical simulations comparing mixing length scales. Ethanol mole fraction within the lipid injection port (-250 μm), at the lipid injection port exit (0 μm), and within the conical mixing chamber (250-1500 μm) is presented for the case of (A) microfluidic vortex focusing and (B) hydrodynamic focusing in the absence of vortex formation.

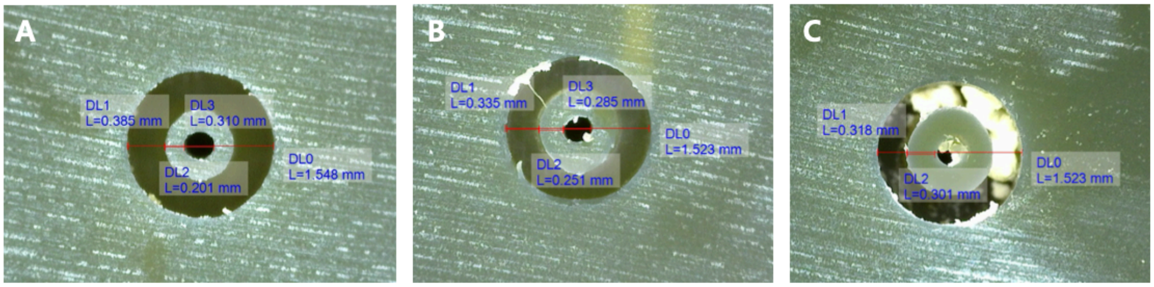

Supplementary Figure 3: Variable SLA-DLP print quality. Example SLA-DLP test structures used for fabrication process optimization depicting (A) successful print with a 300 µm channel, (B) poor print with warping of a 250 µm injection channel, and (C) poor print with partial cloggingof a 250 µm channel.

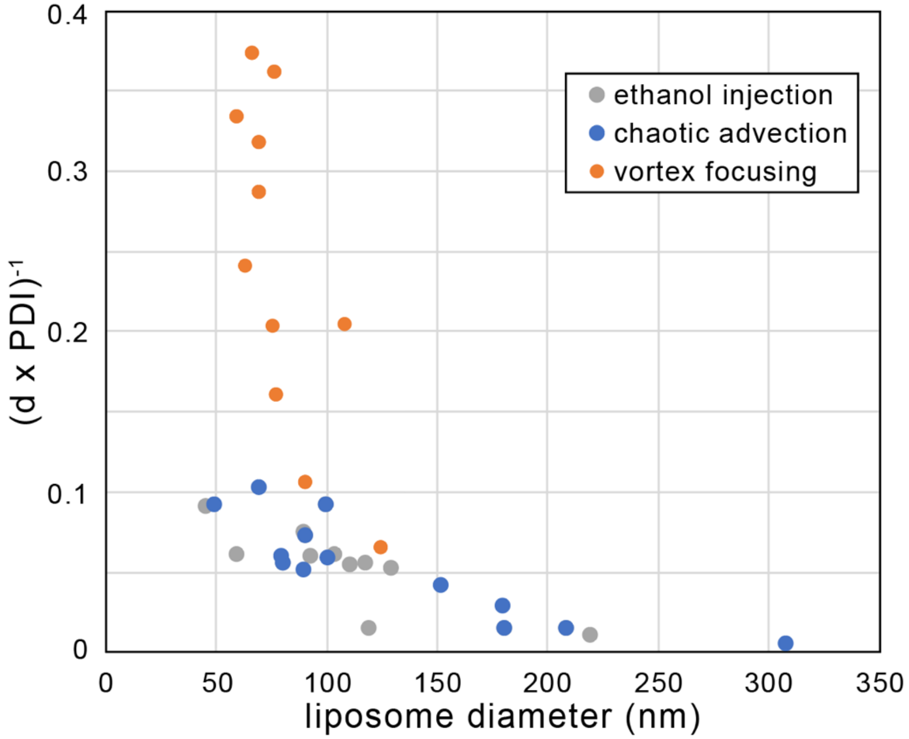

Supplementary Figure 4: Comparison of MVF device performance. Relative performance is presented using a figure of merit defined by the inverse product of vesicle diameter (d) and PDI for liposomes generated by ethanol injection, chaotic advection micromixing, and microfluidic vortex focusing. MVF data is presented in Figures 4 and 5. Ethanol injection and chaotic advection micromixer data are taken from the literature.[25,48,50,80–90] All chaotic advection micromixer data was generated using a commercial platform (NanoAssemblr, Precision NanoSystems Inc., Vancouver, Canada). All presented data is limited to empty liposomes formed without encapsulants using lipid mixtures with fully saturated neutral lipids as the primary component.

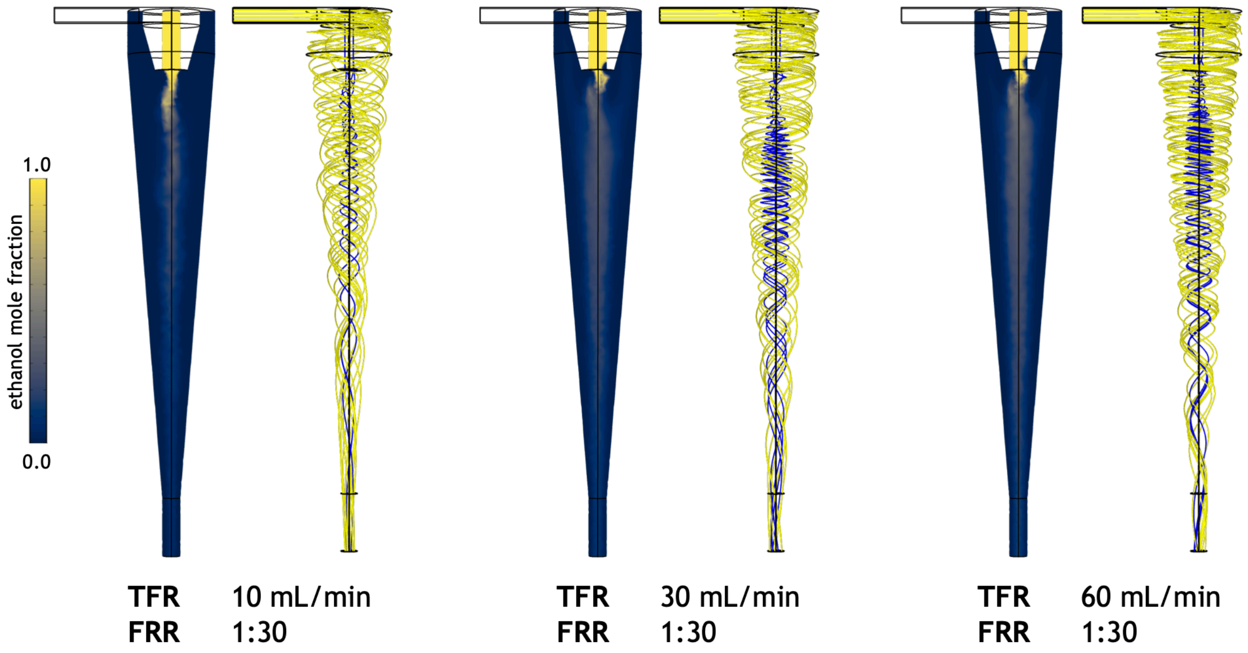

Supplementary Figure 5: Numerical analysis revealing the impact of total flow rate on mixing dynamics. Numerical results depicting solvent concentration profiles and buffer/solvent streamlines while operating the vortex focusing process at total flow rates of 10, 30, and 60 mL/min, with a constant buffer:ethanol flow rate ratio of 30.

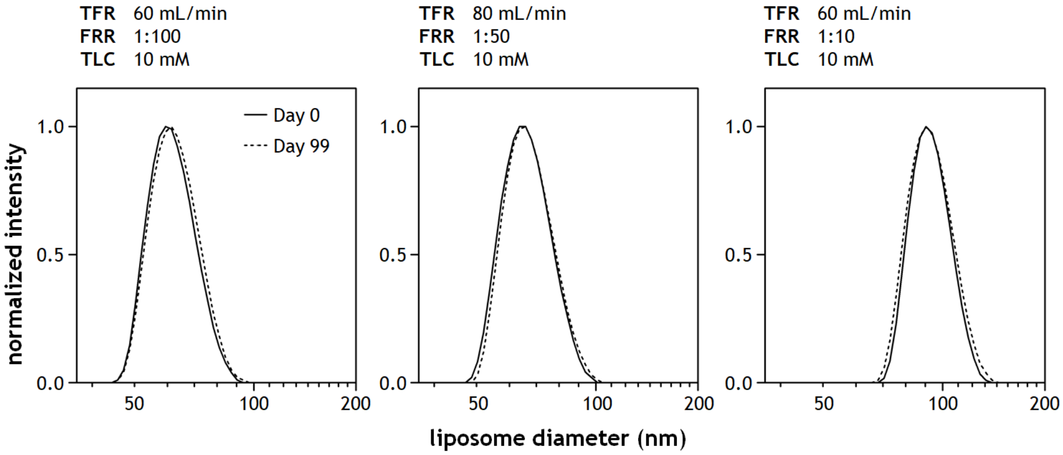

Supplementary Figure 6: Evaluation of long-term colloidal stability of liposomes generated by MVF. Colloidal stability after 99 days storage at 4 °C. for liposomes produced using DMPC:cholesterol:DCP (5:4:1 molar ratio) at selected TFR and FRR values is observed, with negligible change in size distributions over this period.
